# Gut microbiota dysbiosis in sepsis: mechanisms and the gut–organ axis with a focus on lung and brain interactions

**DOI:** 10.3389/fmicb.2026.1797486

**Published:** 2026-07-08

**Authors:** Rui Liu, Junlong Wang

**Affiliations:** 1 Department of Emergency Medicine, The Second Affiliated Hospital of Zhejiang Chinese Medical University, Hangzhou, China; 2 Department of Urology, The First Affiliated Hospital of Zhejiang Chinese Medical University (Zhejiang Provincial Hospital of Chinese Medicine), Hangzhou, China

**Keywords:** fecal microbiota transplantation, gut microbiota, gut-organ axis, metabolomics, pathophysiological mechanisms, precision therapy, sepsis

## Abstract

Sepsis is a life-threatening organ dysfunction caused by a dysregulated host response to infection, with its high mortality closely linked to complex pathophysiological processes. In recent years, the gut microbiota, as the largest human micro-ecosystem, has garnered increasing attention for its critical role in the onset, progression, and prognosis of sepsis. This narrative review summarizes recent research advances, with a particular focus on studies published over the past 3 years, while incorporating selected earlier studies to provide essential mechanistic background. It first delves into the pathophysiological mechanisms underlying sepsis-induced gut microbiota imbalance, highlighting key factors such as intestinal barrier disruption, immune-microbiota interaction disturbances, and alterations in microbial metabolites. Subsequently, the review comprehensively evaluates clinical diagnostic biomarker potentials and therapeutic strategies centered on gut microbiota modulation, including probiotics, prebiotics, fecal microbiota transplantation, and targeted interventions on microbial metabolites. Finally, current research challenges and future translational directions are discussed, aiming to provide novel theoretical foundations and strategic insights for precise prevention and treatment of sepsis. However, most microbiota-targeted therapeutic strategies remain at the preclinical or early clinical stage, and their efficacy and safety in sepsis require further validation.

## 1 Introduction

Sepsis represents a significant global health challenge, characterized as a life-threatening organ dysfunction resulting from a dysregulated host response to infection. It is a leading cause of morbidity and mortality in intensive care units, with estimates suggesting that it accounts for approximately 20% of all deaths worldwide. The pathophysiology of sepsis is complex and multifactorial, involving systemic inflammation, immune suppression, coagulopathy, and multi-organ failure. Traditional research has predominantly focused on the pathogens responsible for sepsis and the host immune response. However, increasing evidence suggests that factors beyond the invading pathogen, including host–microbiome interactions, may substantially influence disease progression and outcomes (Du et al., 2021). Recent advances in our understanding of the gut microbiota have revealed its pivotal role in modulating immune responses and influencing the pathophysiology of sepsis through various organ–gastrointestinal tract axes (Wang et al., 2025).

The gut microbiota, which consists of trillions of microorganisms residing in the human gastrointestinal tract, plays an essential role in maintaining host health. It is involved in numerous physiological processes, including digestion, metabolism, and immune system regulation. Dysbiosis, or an imbalance in the gut microbiota, has been increasingly recognized as a contributing factor to a variety of diseases, including sepsis. Emerging microbiome-based therapeutic strategies have also attracted increasing attention across multiple disease settings. For example, microbiome-directed interventions have been proposed as novel approaches to mitigate chemotherapy-induced mucositis and restore intestinal homeostasis in oncology patients, highlighting the broader translational potential of microbiome-targeted therapies beyond infectious diseases (Obeagu, 2026). Recent studies have shown that sepsis can lead to significant alterations in the gut microbiota, characterized by a decrease in microbial diversity, a reduction in beneficial bacteria, and an overgrowth of opportunistic pathogens (Wang et al., 2025). These changes in the microbiome are not merely a consequence of sepsis; rather, they can exacerbate disease severity and contribute to adverse clinical outcomes, including increased mortality risk (Du et al., 2021).

Emerging evidence suggests that the gut microbiota can influence the immune response during sepsis through several mechanisms. For instance, gut-derived factors such as microbial translocation, endotoxins, and altered metabolite production can exacerbate systemic inflammation and organ injury (Wang et al., 2025). Metabolites produced by gut bacteria, such as short-chain fatty acids, play a crucial role in modulating immune function and maintaining gut barrier integrity. Dysregulation of these metabolites during sepsis may lead to further immunological dysfunction and contribute to multi-organ failure (Lindner et al., 2023). Furthermore, interactions between the gut microbiota and distant organs, particularly the lungs and brain, highlight the complexity of gut–organ communication in sepsis pathophysiology (He et al., 2025; Ye et al., 2025).

Given the critical role of the gut microbiota in sepsis, there is a growing interest in microbiota-targeted therapeutic strategies as adjunctive treatments for sepsis management. Interventions such as probiotics, prebiotics, synbiotics, and fecal microbiota transplantation have shown promise in preclinical studies and early clinical investigations, although robust evidence from large randomized trials remains limited (Klingensmith and Coopersmith, 2023; Niu and Chen, 2021). However, challenges remain regarding patient heterogeneity, safety concerns, and the lack of precise biomarkers for monitoring microbiota changes and therapeutic efficacy. Understanding the underlying mechanisms of gut microbiota dysbiosis in sepsis and developing targeted interventions could significantly improve patient outcomes and reduce mortality associated with this complex condition.

In summary, the interplay between sepsis and gut microbiota dysbiosis represents a rapidly evolving area of research with important mechanistic and clinical implications. A deeper understanding of the pathways linking microbial dysbiosis, intestinal barrier dysfunction, immune dysregulation, and distant organ injury may facilitate the development of novel diagnostic and therapeutic strategies for sepsis. This review aims to provide a narrative synthesis of recent advances in this field, with particular emphasis on evidence published during the past 3 years (2023–2026), while incorporating selected earlier studies that provide essential mechanistic and conceptual foundations.

Several reviews have previously explored the relationship between sepsis and gut microbiota dysbiosis (Klingensmith and Coopersmith, 2023; Niu and Chen, 2021). However, most have focused either on general microbiome alterations or on specific therapeutic interventions. In contrast, the present review provides an updated synthesis of emerging evidence published between 2023 and 2026 and integrates current knowledge across multiple domains, including intestinal barrier dysfunction, immune–microbiota interactions, microbial metabolite alterations, microbiota-based biomarkers, and gut–organ axis communication. Particular emphasis is placed on the gut–lung and gut–brain axes, translational challenges, and the development of precision microbiota-targeted therapeutic strategies. By combining mechanistic, diagnostic, and therapeutic perspectives, this review seeks to provide a comprehensive and clinically relevant framework for understanding the role of gut microbiota in sepsis.

### 1.1 Literature selection strategy

This narrative review was based on a targeted literature search of PubMed/MEDLINE, Web of Science, and Embase databases. Priority was given to studies published between January 2023 and March 2026 that investigated gut microbiota alterations, biomarkers, gut–organ axis interactions, and microbiota-targeted therapeutic strategies in sepsis. Search terms included “sepsis,” “gut microbiota,” “dysbiosis,” “gut–lung axis,” “gut–brain axis,” “metabolomics,” “probiotics,” “prebiotics,” “synbiotics,” and “fecal microbiota transplantation.” Additional studies were identified through manual screening of reference lists from relevant articles. Earlier studies published between 2021 and 2022 were selectively included when they provided foundational mechanistic insights or landmark observations that remain highly relevant to the current understanding of sepsis-associated gut microbiota dysregulation.

## 2 The core pathophysiological mechanisms of sepsis-induced gut microbiota dysregulation

### 2.1 Disruption of intestinal barrier integrity and bacterial/endotoxin translocation

The integrity of the intestinal barrier is essential for maintaining host homeostasis and preventing the translocation of bacteria and microbial products into the systemic circulation. During sepsis, excessive production of inflammatory mediators, including tumor necrosis factor-alpha (TNF-α) and interleukin-6 (IL-6), activates signaling pathways that disrupt intestinal epithelial tight junctions. Degradation of key tight junction proteins, such as occludin and zonula occludens-1 (ZO-1), leads to increased intestinal permeability and compromised barrier integrity (Niu and Chen, 2021). This disruption facilitates the translocation of gut-derived Gram-negative bacteria and lipopolysaccharide (LPS) into the portal and systemic circulation, triggering further inflammatory responses and contributing to organ dysfunction.

In addition to tight junction disruption, dysregulation of epithelial cell homeostasis plays an important role in barrier failure. Excessive epithelial apoptosis, impaired autophagy, and defective mucosal repair mechanisms collectively contribute to the loss of epithelial integrity and increased microbial translocation (Lindner et al., 2023; Ling et al., 2023). The resulting bacterial and endotoxin translocation may act as a “second hit,” amplifying systemic inflammation and establishing a self-perpetuating cycle of intestinal barrier dysfunction and organ injury. Similar mechanisms have also been reported in neonatal sepsis, although extrapolation of these findings to adult populations requires cautious interpretation (Lee and Chiu, 2024).

**FIGURE 1:**
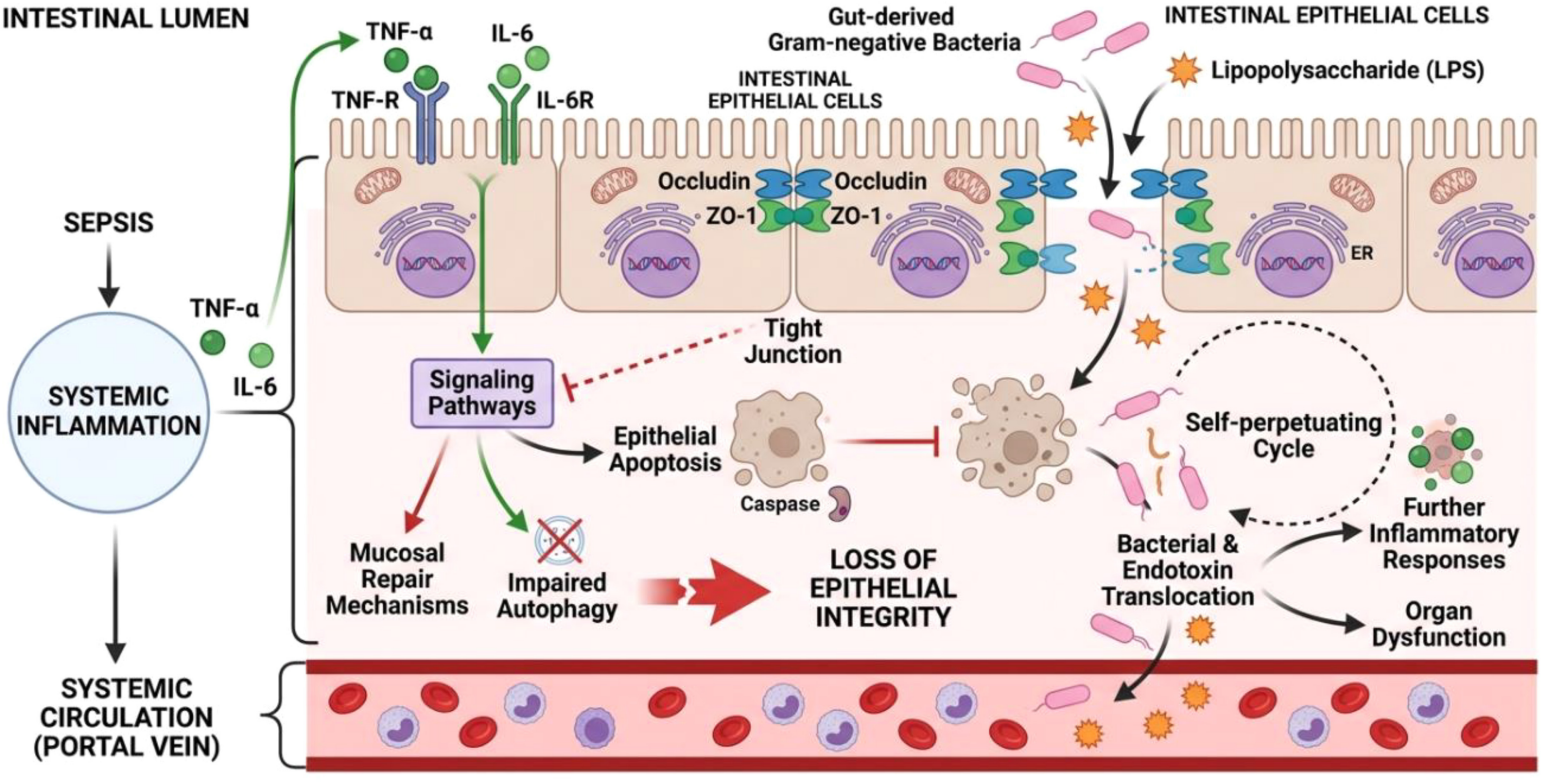
Mechanisms of gut barrier dysfunction and bacterial translocation in sepsis. This figure illustrates the key processes underlying intestinal barrier disruption during sepsis. Systemic inflammation, characterized by increased levels of cytokines such as TNF-α and IL-6, leads to the degradation of tight junction proteins (e.g., occludin and ZO-1), resulting in increased intestinal permeability. Concurrently, dysbiosis of the gut microbiota and alterations in epithelial cell homeostasis (including increased apoptosis and impaired autophagy) further compromise barrier integrity. These changes facilitate the translocation of gut-derived bacteria and microbial products, such as lipopolysaccharide (LPS), into the systemic circulation, contributing to a “second-hit” inflammatory response and promoting multi-organ dysfunction. TNF-α, tumor necrosis factor-alpha; IL-6, interleukin-6; LPS, lipopolysaccharide.

Collectively, inflammatory cytokine-mediated tight junction disruption, epithelial cell injury, and microbial translocation constitute key mechanisms underlying sepsis-induced gut microbiota dysregulation and systemic disease progression (Figure 1).

### 2.2 Dysregulation of the immune system and gut microbiota interactions

Bidirectional interactions between the immune system and the gut microbiota are essential for maintaining intestinal and systemic homeostasis. During sepsis, profound immune dysregulation disrupts this equilibrium and contributes to the development of microbiota dysbiosis. Activation of pattern-recognition receptors (PRRs), particularly Toll-like receptors (TLRs) that recognize pathogen-associated molecular patterns such as lipopolysaccharide (LPS), triggers excessive production of pro-inflammatory cytokines and amplifies systemic inflammatory responses (Shang et al., 2024; Shang et al., 2025). While these responses are initially protective, sustained immune activation may impair tolerance to commensal microorganisms and promote alterations in microbial community composition.

As sepsis progresses, the host immune response frequently shifts from an early hyper-inflammatory state toward a phase characterized by immune dysfunction and immunosuppression. This transition is associated with impaired antigen presentation, lymphocyte depletion, altered T-cell differentiation, and reduced immune surveillance, which collectively increase susceptibility to secondary infections and adverse clinical outcomes (Shang et al., 2025). Concurrently, gut microbiota dysbiosis further exacerbates immune dysfunction through reduced production of immunoregulatory microbial metabolites and disruption of host–microbe signaling pathways (Xu et al., 2022).

**FIGURE 2:**
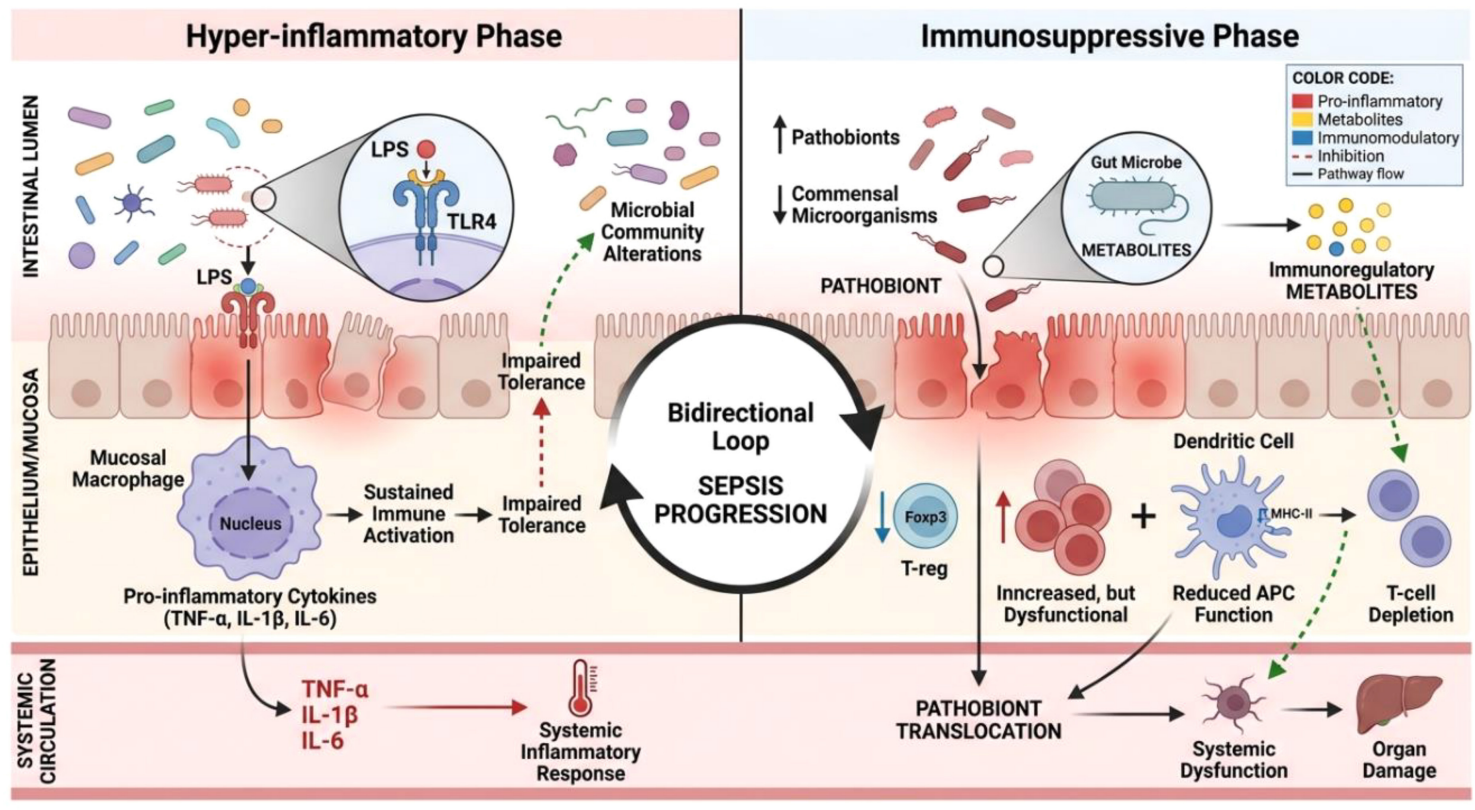
Dysregulation of the immune system and gut microbiota interactions in sepsis. This figure illustrates the bidirectional interactions between gut microbiota dysbiosis and immune dysfunction during sepsis. Disruption of the gut microbial community leads to reduced production of beneficial metabolites, such as short-chain fatty acids (SCFAs), and increased release of pathogen-associated molecular patterns (PAMPs), including lipopolysaccharide (LPS). These signals activate innate immune pathways, promoting excessive inflammatory responses characterized by elevated cytokine production (e.g., TNF-α, IL-6). At the same time, sepsis is associated with immune dysregulation, including impaired antigen presentation, T cell exhaustion, and reduced immune surveillance. These changes further exacerbate microbiota imbalance and compromise intestinal barrier integrity, creating a vicious cycle between dysbiosis and immune dysfunction. Overall, this figure highlights the reciprocal relationship between gut microbiota alterations and immune system dysregulation, which contributes to the progression and severity of sepsis. SCFAs, short-chain fatty acids; PAMPs, pathogen-associated molecular patterns; LPS, lipopolysaccharide; TNF-α, tumor necrosis factor-alpha; IL-6, interleukin-6.

The relationship between immune dysfunction and gut microbiota alterations is therefore bidirectional. Immune dysregulation promotes microbial imbalance, whereas microbiota dysbiosis further impairs immune homeostasis, creating a self-reinforcing cycle that contributes to disease progression and organ dysfunction during sepsis. Understanding these complex host–microbiome interactions may facilitate the development of novel immunomodulatory and microbiota-targeted therapeutic strategies (Figure 2).

Importantly, intestinal barrier dysfunction, immune dysregulation, and microbial metabolic alterations should not be viewed as independent processes. Rather, they constitute an interconnected pathogenic network. Increased intestinal permeability facilitates microbial translocation and immune activation, while dysregulated immune responses further aggravate epithelial injury and alter microbial composition. Concurrently, loss of beneficial microbial metabolites weakens barrier integrity and impairs immune homeostasis, thereby amplifying systemic inflammation and promoting distant organ dysfunction. Together, these processes establish a self-reinforcing cycle that contributes to the progression of sepsis.

### 2.3 Altered gut microbiota metabolic profiles and systemic impacts

Sepsis not only disrupts the composition of the gut microbiota but also profoundly alters its metabolic activity, resulting in systemic consequences that may influence disease progression and outcomes. One of the most consistently reported metabolic alterations is the depletion of short-chain fatty acids (SCFAs), including butyrate, propionate, and acetate, which are generated through microbial fermentation of dietary fibers (Lindner et al., 2023). SCFAs play essential roles in maintaining intestinal barrier integrity, regulating immune responses, and serving as energy substrates for colonic epithelial cells. Consequently, the loss of SCFA-producing bacteria during sepsis contributes to reduced metabolite availability, increased intestinal permeability, and exaggerated inflammatory responses (Niu and Chen, 2021).

In addition to SCFA depletion, alterations in tryptophan metabolism have emerged as another important feature of microbiota-associated metabolic dysregulation. Increased flux through the kynurenine pathway may influence immune regulation, neuroinflammatory signaling, and gut–brain communication during sepsis. Although some evidence derives from neonatal populations, these findings suggest a potentially important role for tryptophan metabolites in host–microbiome interactions during sepsis (Lee and Chiu, 2024).

Dysregulation of bile acid metabolism also contributes to the systemic metabolic consequences of gut microbiota dysbiosis. Because intestinal microorganisms are critical regulators of primary and secondary bile acid transformation, alterations in microbial composition can affect bile acid signaling pathways involved in glucose homeostasis, immune regulation, and inflammatory responses (Chen et al., 2025). Emerging evidence further suggests that microbiota-derived metabolites participate in host immunometabolic reprogramming and may influence the development of organ dysfunction during sepsis.

**FIGURE 3:**
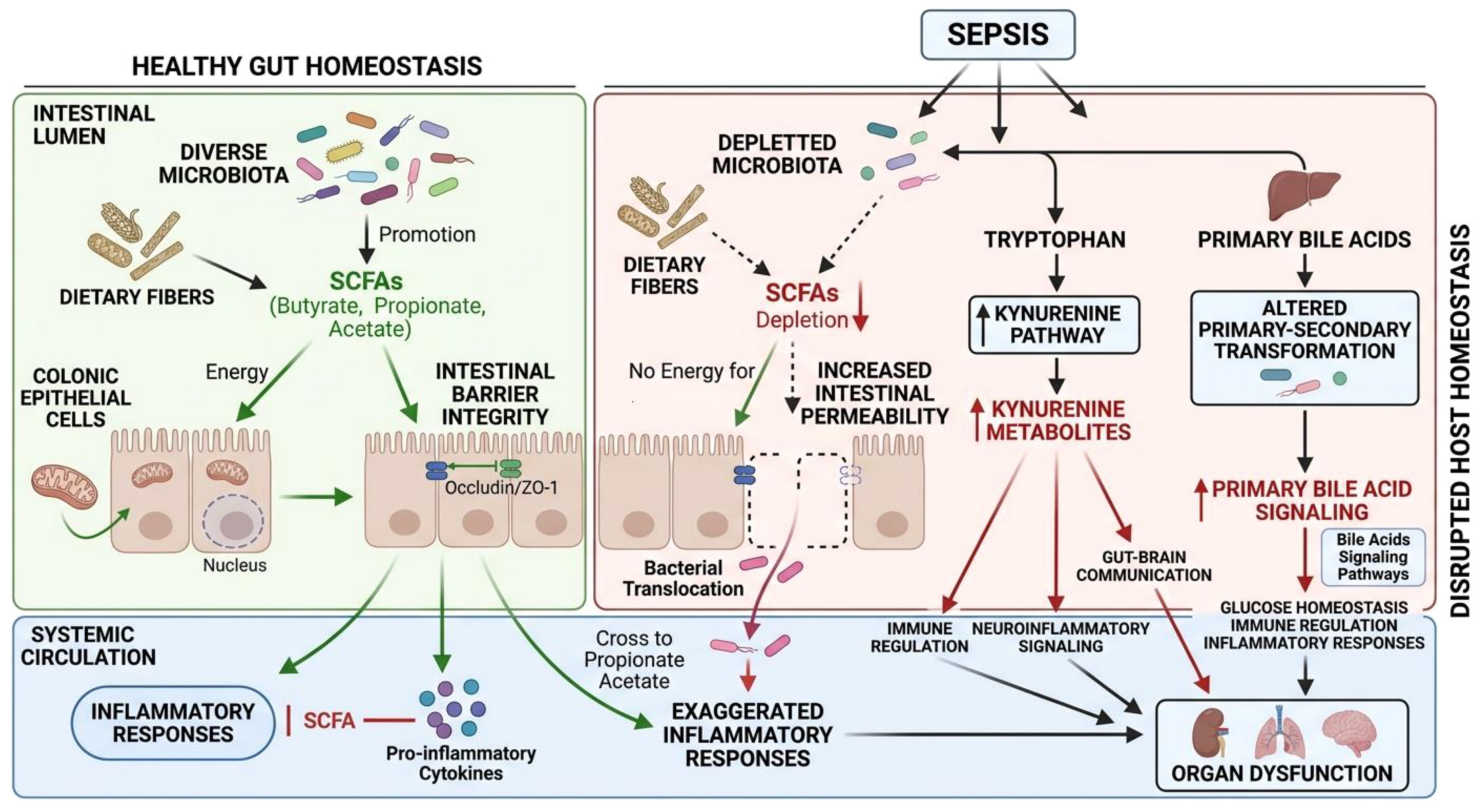
Microbiota-derived metabolic alterations in sepsis. This figure summarizes key changes in gut microbiota-derived metabolites during sepsis. A reduction in short-chain fatty acids (SCFAs), including butyrate, propionate, and acetate, contributes to impaired epithelial barrier function and dysregulated immune responses. Alterations in tryptophan metabolism, particularly increased flux through the kynurenine pathway, may affect gut–brain communication and immune modulation. In addition, dysregulated bile acid metabolism may influence systemic inflammation and metabolic homeostasis. Overall, these metabolic disturbances highlight the role of the gut microbiota as a regulator of host immune and metabolic pathways during sepsis. Abbreviations: SCFAs, short-chain fatty acids.

Collectively, these metabolic disturbances underscore the central role of the gut microbiota as a regulator of host metabolic and immune homeostasis. Restoration of microbial metabolic function may therefore represent a promising therapeutic strategy for improving outcomes in sepsis (Figure 3).

Microbiota-derived metabolites may exert either protective or detrimental effects during sepsis. SCFAs, secondary bile acids, and selected indole derivatives generally support epithelial barrier integrity, immune homeostasis, and anti-inflammatory signaling. In contrast, elevated concentrations of trimethylamine N-oxide (TMAO), a metabolite generated from dietary choline and carnitine through microbial metabolism, have been associated with endothelial dysfunction, oxidative stress, and adverse cardiovascular outcomes. Although direct evidence in sepsis remains limited, these observations highlight the diverse biological effects of microbiota-derived metabolites and suggest that individual metabolites should be evaluated separately when developing biomarkers or microbiota-targeted therapeutic strategies.

## 3 The potential of gut microbiota as biomarkers for sepsis

### 3.1 Predictive models based on microbiota composition and diversity

The gut microbiota has emerged as a promising source of biomarkers for risk stratification and prognostic assessment in sepsis. Recent clinical studies have shown that sepsis is associated with marked alterations in gut microbial diversity, community structure, and taxonomic composition, and that these changes may correlate with disease severity, treatment course, and survival outcomes (Liu et al., 2023; Luan et al., 2024; Sun et al., 2023; Wozniak et al., 2024).

Several observational studies have reported reduced microbial diversity during critical illness or sepsis. Dynamic analyses of septic patients have shown that gut microbiota diversity may decrease during ICU stay, accompanied by expansion of potentially pathogenic taxa such as Enterococcus (Liu et al., 2023). More recent prospective evidence further suggests that early reduction in gut microbiota diversity is associated with increased mortality in critically ill patients (Wozniak et al., 2024). These findings support the potential value of microbiota diversity indices, including Shannon diversity and related metrics, as prognostic indicators, although validation in larger multicenter cohorts remains necessary.

Beyond overall diversity, specific microbial taxa and compositional signatures may also serve as candidate biomarkers. A multicenter study of patients with sepsis found dynamic changes in gut microbiota composition during the early phase of sepsis and suggested that changes in the abundance of specific taxa may help predict survival (Luan et al., 2024). In addition, integrated microbiome and metabolome profiling has demonstrated that altered intestinal microbial communities and metabolite patterns correspond to different clinical outcomes in sepsis (Sun et al., 2023). These findings indicate that microbiota-based biomarkers may reflect not only local intestinal dysbiosis but also broader host metabolic and inflammatory responses.

Importantly, critical illness is characterized by substantial interindividual variation in microbiota disruption. Longitudinal analyses have shown that gut microbiome dynamics are associated with mortality in critically ill patients, supporting the need for repeated sampling and dynamic monitoring rather than single time-point assessment (Salameh et al., 2023). Furthermore, dysbiosis of the microbiota–immune metasystem has been linked to nosocomial infections in critical illness, highlighting the potential role of microbiota profiling in identifying patients at risk for secondary infectious complications (Schlechte et al., 2023). Collectively, these studies suggest that future predictive models may integrate microbial diversity indices, taxonomic signatures, metabolomic features, and host immune parameters to improve risk stratification and prognostic assessment in sepsis.

### 3.2 Biomarkers based on microbiota functional genes and metabolites

In addition to compositional analysis, functional metagenomics and metabolomics provide deeper insights into the metabolic capabilities of the gut microbiota during sepsis. Recent microbiome–metabolome studies have shown that sepsis is associated with disruption of microbial metabolic pathways, including short-chain fatty acid (SCFA)-related metabolism, amino acid metabolism, tryptophan metabolism, and other host–microbiome co-metabolic processes (Li et al., 2023; Wang et al., 2024). Conversely, enrichment of pathways related to endotoxin production, virulence factors, and antimicrobial resistance may contribute to excessive inflammation, immune dysregulation, and adverse clinical outcomes (Wang et al., 2024).

Compared with taxonomic composition alone, functional microbial signatures and metabolite profiles may provide more biologically informative indicators of host–microbiome interactions. Integrated microbiome and metabolome analyses have demonstrated that altered intestinal microbial communities and metabolic features correspond to different clinical outcomes in sepsis (Wang et al., 2024). In parallel, metabolomics-based studies have identified candidate metabolic biomarkers that may help distinguish sepsis from non-septic conditions and support early diagnostic assessment (Li et al., 2023).

Specific microbiota-related metabolites, including SCFAs, tryptophan-derived metabolites, amino acid metabolites, and bile acid-related metabolic signatures, have been associated with systemic inflammation, organ dysfunction, and mortality risk in sepsis (Feng et al., 2023; Jin et al., 2025; Pandey, 2025; Yagin et al., 2024). Some metabolite panels have also been proposed for mortality prediction and disease monitoring using liquid chromatography–mass spectrometry-based approaches (Feng et al., 2023). Although these findings remain exploratory and require external validation, functional microbial genes and microbiota-derived metabolites represent promising candidates for next-generation sepsis biomarkers.

**FIGURE 4:**
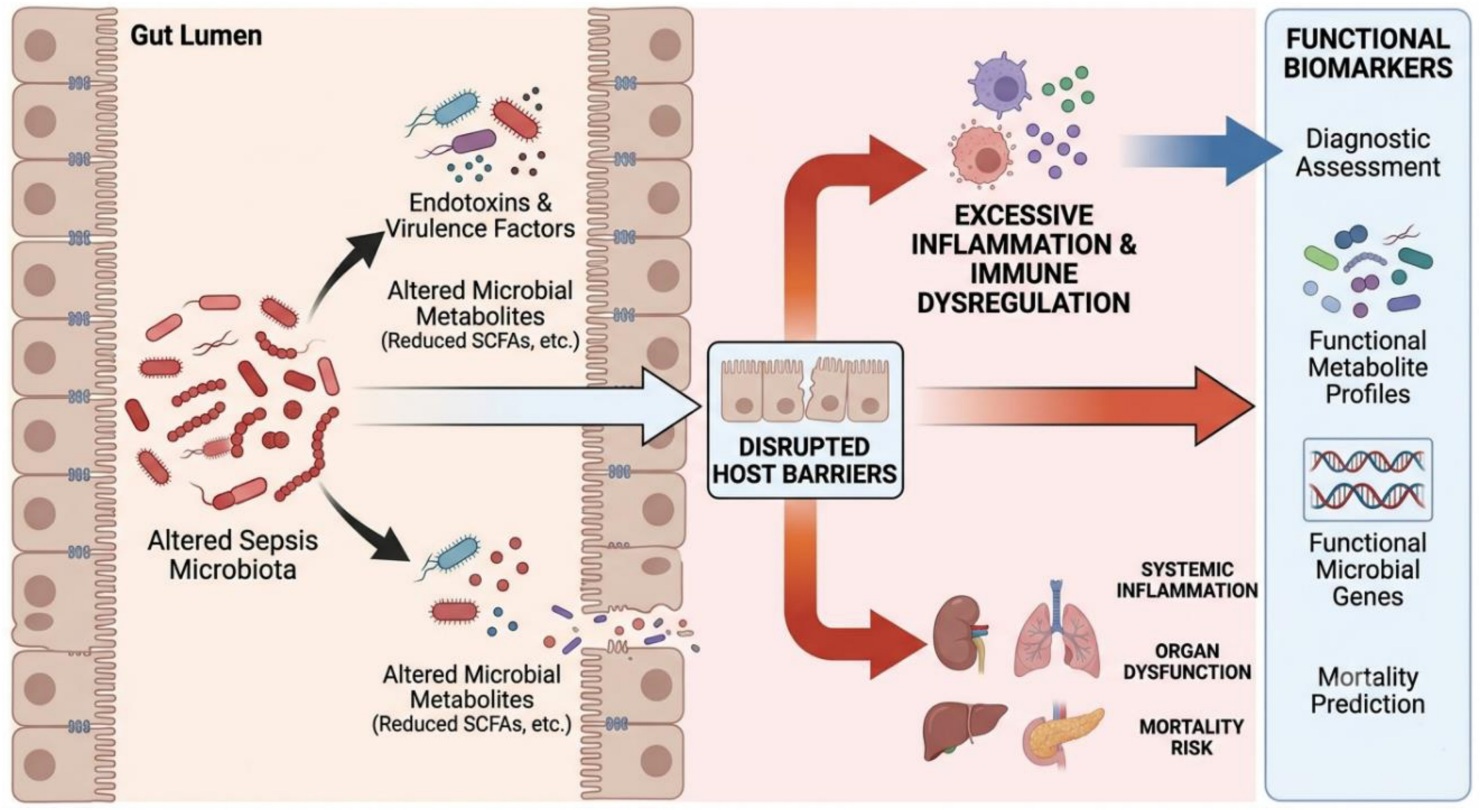
The potential of gut microbiota as biomarkers for sepsis. This figure summarizes the potential application of gut microbiota-derived features as biomarkers for sepsis. Changes in microbial composition (e.g., reduced diversity and enrichment of pathogenic taxa), functional gene profiles, and metabolite signatures (such as SCFAs and bile acids) may reflect disease severity and host–microbiome interactions. These microbiota-associated indicators may contribute to risk stratification, prognosis assessment, and monitoring of disease progression. However, most evidence is derived from observational studies, and significant challenges remain, including inter-individual variability, lack of standardization in sampling and analysis, and limited validation in large clinical cohorts. Overall, while gut microbiota-based biomarkers show promise, their clinical utility in sepsis remains to be established and requires further validation. SCFAs, short-chain fatty acids.

Future studies integrating metagenomics, metabolomics, transcriptomics, machine learning, and host immune profiling may facilitate the development of robust microbiota-based diagnostic and prognostic tools for sepsis (Jin et al., 2025; Pandey, 2025; Prucha et al., 2018; Yagin et al., 2024; Figure 4). Representative human studies on microbiota-based biomarkers in sepsis are summarized in Table 1.

**TABLE 1:** Representative clinical studies investigating microbiota-associated biomarkers in sepsis.

Study	Population	Sample type	Biomarker/ feature	Main findings	Clinical relevance	Evidence type
Luan et al. (2024)	Adult sepsis patients	Stool	Gut microbiota composition and diversity	Dynamic alterations in microbial composition were associated with survival outcomes	Potential prognostic biomarker	Prospective observational study
Liu et al. (2023)	Adult sepsis patients	Stool	Microbial diversity and taxonomic signatures	Reduced microbial diversity and Enterococcus enrichment correlated with disease severity	Severity assessment and risk stratification	Observational study
Sun et al. (2023)	Adult sepsis patients	Stool and plasma	Integrated microbiome–metabolome profiles	Distinct microbial and metabolic signatures corresponded to different clinical outcomes	Prognostic biomarker panel	Multi-omics observational study
Wozniak et al. (2024)	Critically ill patients	Stool	Alpha diversity	Early reduction in gut microbiota diversity was associated with increased mortality	Prognostic biomarker	Prospective cohort study
Salameh et al. (2023)	Critically ill patients	Stool	Gut microbiome dynamics	Longitudinal microbiome changes correlated with survival and clinical outcomes	Dynamic monitoring biomarker	Longitudinal cohort study
Schlechte et al. (2023)	ICU patients	Stool and blood	Microbiota–immune metasystem signatures	Dysbiosis was associated with nosocomial infections and adverse clinical outcomes	Risk stratification and infection prediction	Prospective cohort study
Wang et al. (2024)	Adult sepsis patients	Stool and plasma	Gut microbiota–metabolite signatures	Alterations in gut microbiota and metabolites were associated with disrupted vitamin metabolism during sepsis	Functional microbiome biomarker	Multi-omics observational study

These studies demonstrate that alterations in gut microbial diversity, taxonomic composition, microbiota–immune interactions, and microbiome-associated metabolic signatures are associated with disease severity, survival, nosocomial infections, and clinical outcomes in patients with sepsis or critical illness. Although these findings support the potential utility of microbiota-associated biomarkers for diagnosis, prognosis, and risk stratification, most evidence remains observational and requires validation in larger multicenter cohorts.

## 4 Targeting gut microbiota: new strategies for sepsis treatment

### 4.1 Microbial preparations: probiotics, prebiotics, and synbiotics

#### 4.1.1 Efficacy and mechanisms of specific strains

Recent randomized controlled trials (RCTs), systematic reviews, and meta-analyses have produced mixed results regarding the efficacy of conventional probiotics, including *Lactobacillus* and Bifidobacterium species, for the prevention and treatment of sepsis (Manzanares et al., 2016; Wischmeyer et al., 2023). While some studies have reported reductions in infectious complications and improvements in gastrointestinal barrier function, others have failed to demonstrate consistent survival benefits, highlighting substantial heterogeneity among patient populations, probiotic formulations, and treatment protocols.

Increasing attention has therefore shifted toward next-generation probiotics and targeted microbiota-based interventions. Experimental studies have identified butyrate-producing bacteria, including *Faecalibacterium prausnitzii*, as potential therapeutic candidates because of their ability to enhance intestinal barrier integrity, suppress excessive inflammatory responses, and promote immune homeostasis (Martín et al., 2017; Sokol et al., 2008). Similarly, *Akkermansia muciniphila* has demonstrated protective effects in preclinical models through reinforcement of the mucus layer, modulation of host metabolism, and regulation of mucosal immune responses (Depommier et al., 2019).

The mechanisms underlying these beneficial effects are multifactorial. Probiotics and synbiotics may reduce intestinal permeability, strengthen epithelial tight junctions, increase production of short-chain fatty acids (SCFAs), regulate innate and adaptive immune responses, and inhibit colonization by opportunistic pathogens (Manzanares et al., 2016; Martín et al., 2017; McDonald et al., 2016). Through these pathways, microbiota-directed therapies may attenuate systemic inflammation and mitigate organ dysfunction during sepsis.

Although clinical evidence remains insufficient to support routine implementation of next-generation probiotics in septic patients, these approaches represent an important area of ongoing research. Future studies should focus on strain-specific efficacy, patient stratification, safety considerations, and integration with precision microbiome medicine strategies (Depommier et al., 2019; McDonald et al., 2016; Wischmeyer et al., 2023).

#### 4.1.2 Precision application of prebiotics and synbiotics

The precision application of prebiotics and synbiotics represents an emerging strategy for microbiota modulation in sepsis and critical illness. Specific prebiotic substrates, including inulin, resistant starch, galacto-oligosaccharides, and other fermentable fibers, selectively promote beneficial commensal bacteria and enhance the production of short-chain fatty acids (SCFAs), thereby supporting intestinal barrier integrity, microbial homeostasis, and immune regulation (Gibson et al., 2017; Tan et al., 2014; Wischmeyer et al., 2023). Studies in critically ill populations suggest that prebiotic-enriched enteral nutrition may partially restore gut microbial diversity and improve intestinal ecosystem stability, although evidence specifically derived from septic patients remains limited (Lou et al., 2024; Wischmeyer et al., 2023).

Synbiotics, which combine probiotics and prebiotics, have shown promise in several clinical studies and meta-analyses involving critically ill patients. Reported benefits include modulation of gut microbiota composition, enhancement of mucosal barrier function, and reduction of certain infection-related complications. However, the available evidence remains heterogeneous because of differences in probiotic strains, synbiotic formulations, treatment duration, and patient populations (Bagdadi et al., 2025; Chotiprasitsakul et al., 2023; Lou et al., 2024; Manzanares et al., 2016; Wischmeyer et al., 2023). Consequently, the efficacy of prebiotics and synbiotics in sepsis remains inconclusive and requires confirmation in adequately powered, sepsis-specific randomized controlled trials.

Despite encouraging preclinical findings, the clinical efficacy of probiotics in sepsis remains inconsistent, likely owing to several important factors. First, strain specificity appears to be critical. Most clinical studies have evaluated conventional *Lactobacillus*- and Bifidobacterium-based formulations, whereas next-generation commensals such as *Faecalibacterium prausnitzii* and *Akkermansia muciniphila* possess more specialized anti-inflammatory, barrier-protective, and immunometabolic functions but are rarely incorporated into clinical trials (Depommier et al., 2019; Martín et al., 2017). Second, baseline microbiota heterogeneity among septic patients may substantially influence treatment response. Prior antibiotic exposure, nutritional status, organ dysfunction, and critical illness-associated dysbiosis can alter microbial colonization resistance and reduce the effectiveness of administered microbial preparations (Shimizu et al., 2025; Wischmeyer et al., 2023; Zmora et al., 2018).

Third, the timing of intervention may be a key determinant of efficacy. Early microbiota-directed therapy during the hyper-inflammatory phase may help preserve epithelial barrier integrity and limit microbial translocation, whereas delayed intervention during established immunosuppression may be less effective in restoring microbiota balance and immune competence (Ling et al., 2023; Lou et al., 2024; Manzanares et al., 2016; Shimizu et al., 2025). Finally, methodological heterogeneity across studies—including differences in dosage, treatment duration, formulation, route of administration, and outcome definitions—continues to complicate interpretation of clinical findings (Bagdadi et al., 2025; Lou et al., 2024; Wischmeyer et al., 2023).

Collectively, these observations suggest that future clinical trials should move beyond conventional probiotic supplementation toward precision microbiota-based strategies. Functional strain selection, microbiota-guided patient stratification, standardized synbiotic formulations, and timing-specific intervention protocols may improve therapeutic efficacy. Integration of microbiome sequencing, metabolomics, and host immune profiling may further facilitate individualized microbiota-targeted interventions for patients with sepsis (Wischmeyer et al., 2023; Zmora et al., 2018).

### 4.2 Fecal microbiota transplantation (FMT): exploration and challenges

#### 4.2.1 Robust evidence from preclinical studies

Accumulating preclinical evidence suggests that fecal microbiota transplantation (FMT) may represent a promising microbiota-directed therapeutic strategy for sepsis. In experimental sepsis models, transplantation of microbiota from healthy donors has been shown to restore microbial diversity, increase the abundance of beneficial commensals, enhance short-chain fatty acid (SCFA) production, improve intestinal barrier integrity, attenuate systemic inflammation, and reduce multi-organ injury (Gai et al., 2021; Han et al., 2024; Ling et al., 2023; Shimizu et al., 2006). Several studies have further demonstrated improved survival in septic animals receiving FMT compared with untreated controls, suggesting that restoration of complex microbial communities may provide advantages over single-strain probiotic supplementation (Gai et al., 2021; Han et al., 2024). These findings support the concept that microbial ecosystem reconstruction may influence host immune responses, metabolic homeostasis, and organ function during sepsis.

#### 4.2.2 Preliminary clinical evidence and safety considerations

Clinical evidence supporting FMT in sepsis remains extremely limited. Available data are largely derived from case reports, pilot studies, and patients with recurrent *Clostridioides difficile* infection complicated by sepsis or critical illness rather than from dedicated sepsis cohorts (Allegretti et al., 2019; Ooijevaar et al., 2019). Although preliminary observations suggest that FMT may facilitate restoration of microbiota composition and recovery of microbial metabolic function, its efficacy and safety in septic patients have not yet been established in adequately powered clinical trials.

Safety remains a major concern, particularly in critically ill and immunocompromised populations. Potential risks include transmission of multidrug-resistant organisms (MDROs), bloodstream infection, microbial translocation, and unpredictable host–microbiome interactions (Cammarota et al., 2019; DeFilipp et al., 2019). Regulatory safety alerts have reported severe infections associated with donor-derived resistant organisms following FMT, highlighting the necessity of rigorous donor screening and quality-control procedures (DeFilipp et al., 2019). Consequently, current expert recommendations emphasize comprehensive donor screening, pathogen surveillance, antimicrobial resistance gene assessment, and standardized manufacturing procedures before clinical implementation (Cammarota et al., 2019; Feuerstadt et al., 2022).

#### 4.2.3 Future directions for clinical translation

From a translational and pharmacological perspective, the implementation of FMT in sepsis requires a structured risk–benefit assessment framework. Potential benefits include restoration of microbial diversity, recovery of metabolite production, reinforcement of intestinal barrier function, and modulation of dysregulated immune responses. However, these benefits must be balanced against substantial safety and regulatory challenges (Cammarota et al., 2019; DeFilipp et al., 2019; Feuerstadt et al., 2022).

Future development should move beyond crude stool transplantation toward more standardized microbiota-based therapeutics, including defined microbial consortia, live biotherapeutic products (LBPs), and precision microbiome interventions (Feuerstadt et al., 2022; Khanna et al., 2016). Standardization of donor selection, manufacturing processes, dosing strategies, administration routes, and patient stratification will be essential to improve reproducibility and safety. At present, FMT should be regarded as an investigational therapy in sepsis that requires further validation through high-quality randomized controlled trials and regulatory oversight before widespread clinical adoption.

### 4.3 Targeting microbial metabolites: interventions

#### 4.3.1 Exogenous SCFA supplementation

Microbiota-derived short-chain fatty acids (SCFAs), particularly butyrate, have emerged as promising therapeutic targets in sepsis. Experimental studies have demonstrated that exogenous butyrate administration can partially reproduce the protective effects of beneficial commensal bacteria by enhancing epithelial barrier integrity, suppressing excessive inflammatory responses, and improving intestinal immune homeostasis (He et al., 2024; Koh et al., 2016; Zhang Q. et al., 2023; Zhang L. et al., 2023). In murine sepsis models, butyrate supplementation has been associated with reduced intestinal permeability, attenuation of systemic inflammation, and improved survival outcomes (Zhang Q. et al., 2023; Zhang L. et al., 2023). These findings suggest that oral butyrate formulations, pro-drugs, or postbiotic approaches may represent attractive translational strategies for microbiota-targeted therapy in sepsis.

#### 4.3.2 Modulation of bile acid metabolism

Bile acids are increasingly recognized as key mediators of microbiota–host communication. Beyond their classical role in lipid metabolism, bile acids regulate immune responses and intestinal homeostasis through activation of the farnesoid X receptor (FXR) and Takeda G protein-coupled receptor 5 (TGR5) signaling pathways (Fiorucci and Distrutti, 2015; Shahid et al., 2024). Experimental studies have shown that modulation of bile acid metabolism, including administration of ursodeoxycholic acid and other secondary bile acids, may attenuate systemic inflammation, improve gut barrier function, and alleviate sepsis-associated organ injury (Agus et al., 2018; Chen et al., 2025; Shahid et al., 2024). These observations highlight bile acid signaling as a promising therapeutic target linking microbial metabolism and host immune regulation.

#### 4.3.3 Supplementation of AhR ligands and other microbial metabolites

Aryl hydrocarbon receptor (AhR) signaling represents another important pathway through which microbial metabolites influence host physiology. Microbiota-derived indole derivatives generated from tryptophan metabolism can activate AhR signaling, promote epithelial regeneration, strengthen barrier integrity, and regulate innate and adaptive immune responses (Krautkramer et al., 2021; Lavelle and Sokol, 2020; Zelante et al., 2013). Preclinical studies suggest that supplementation with AhR agonists, including indole-derived metabolites, may protect against sepsis-induced intestinal injury and immune dysregulation (Krautkramer et al., 2021; Lavelle and Sokol, 2020). In addition to AhR ligands, emerging postbiotic approaches involving defined microbial metabolites are being actively explored as potentially safer and more standardized alternatives to live microbial therapies (Aguilar-Toalá et al., 2021).

Collectively, these findings support the concept that microbial metabolites are not merely biomarkers but also potential therapeutic agents. Future studies should focus on optimizing dosing strategies, improving pharmacokinetic properties, identifying responsive patient populations, and integrating metabolite-based interventions into precision microbiome medicine frameworks. A summary of microbiota-targeted therapeutic strategies in sepsis is provided in Table 2.

**TABLE 2:** Summary of microbiota-targeted therapeutic strategies for sepsis.

Therapeutic strategy	Representative intervention	Proposed mechanisms	Evidence level	Current limitations
Conventional probiotics	*Lactobacillus* spp., *Bifidobacterium* spp.	Modulation of gut microbiota, enhancement of barrier integrity, immune regulation	Preclinical studies, small clinical trials, meta-analyses	Heterogeneous efficacy; strain specificity; limited sepsis-specific evidence
Prebiotics	Inulin, resistant starch, galacto-oligosaccharides	Promotion of beneficial bacteria and SCFA production	Preclinical studies; limited clinical evidence	Variable response; lack of sepsis-specific RCTs
Synbiotics	Combined probiotic–prebiotic formulations	Synergistic microbiota modulation and barrier protection	Small clinical studies; meta-analyses in critical illness	Heterogeneous formulations and outcomes
Next-generation probiotics	*Faecalibacterium prausnitzii, Akkermansia muciniphila*	Anti-inflammatory effects; immunometabolic regulation; enhancement of mucosal integrity	Preclinical evidence	Limited human studies; manufacturing challenges
Fecal microbiota transplantation (FMT)	Healthy donor stool transplantation	Restoration of microbial diversity and metabolic function	Strong preclinical evidence; limited clinical evidence	Safety concerns; donor screening; regulatory challenges
Defined microbial consortia/Live biotherapeutic products	SER-109 and related microbiome therapeutics	Standardized microbiota restoration with improved safety	Early clinical development	Limited sepsis-specific studies
SCFA supplementation	Butyrate, acetate, propionate	Barrier protection; anti-inflammatory effects; immune regulation	Preclinical evidence	Pharmacokinetic and formulation challenges
Bile acid-targeted therapy	Ursodeoxycholic acid; FXR/TGR5 modulators	Regulation of immune responses and metabolic homeostasis	Experimental studies	Lack of clinical validation
AhR ligand supplementation	Indole derivatives; tryptophan metabolites	Activation of AhR signaling; maintenance of barrier integrity; immune modulation	Preclinical evidence	Optimal compounds and dosing remain unclear
Postbiotics	Defined microbial metabolites and bioactive products	Reproducible microbiome-mediated therapeutic effects without live organisms	Emerging preclinical and translational evidence	Limited clinical experience
Precision microbiome medicine	Microbiota-guided patient stratification and targeted interventions	Personalized microbiome modulation based on host–microbiome profiles	Conceptual framework; early translational studies	Requires biomarker validation and prospective trials

Although microbiota-derived metabolites such as short-chain fatty acids (SCFAs), bile acids, and aryl hydrocarbon receptor (AhR) ligands represent promising therapeutic targets, their translation into clinical drug development faces several important challenges (Aguilar-Toalá et al., 2021; Fiorucci and Distrutti, 2015; He et al., 2024; Koh et al., 2016; Krautkramer et al., 2021; Lavelle and Sokol, 2020; Shahid et al., 2024; Zelante et al., 2013). Taking butyrate as an example, its therapeutic application is constrained by poor oral bioavailability, rapid metabolism, and a relatively short systemic half-life. These pharmacokinetic limitations necessitate the development of prodrugs, encapsulation technologies, or targeted delivery systems, such as colon-targeted formulations, to achieve effective local or systemic concentrations (He et al., 2024; Koh et al., 2016). Similarly, bile acid-based therapies require careful optimization because excessive activation of FXR and TGR5 signaling pathways may result in unintended metabolic consequences, including disturbances in lipid and glucose homeostasis (Fiorucci and Distrutti, 2015; Shahid et al., 2024).

In addition, current therapeutic approaches have largely focused on exogenous supplementation of microbial metabolites, whereas comparatively less attention has been directed toward endogenous regulation of microbial metabolic pathways. Modulation of key microbial enzymes, microbial metabolic networks, or host–microbiome co-metabolic pathways may provide a more controllable and pharmacologically tractable strategy for therapeutic intervention (Krautkramer et al., 2021; Lavelle and Sokol, 2020). Furthermore, substantial challenges remain regarding target validation, dose optimization, safety assessment, and interindividual variability in microbiome composition and metabolic responses (Lavelle and Sokol, 2020; Shahid et al., 2024).

Overall, successful translation of microbiota-derived metabolites into clinically applicable therapies will require integration of microbiome science with traditional drug development principles, including pharmacokinetic characterization, formulation engineering, target validation, and biomarker-guided patient selection (Aguilar-Toalá et al., 2021). To further contextualize these challenges and highlight the translational differences among microbiota-targeted interventions, a comparative summary from a drug development perspective is presented in Table 3.

**TABLE 3:** Comparison of microbiota-targeted therapeutic strategies from a drug development perspective.

Strategy	Representative examples	Main advantages	Key challenges	Translational readiness*
Conventional probiotics	*Lactobacillus* spp., *Bifidobacterium* spp.	Established safety profile; commercially available; relatively low cost	Strain-specific efficacy; inconsistent clinical outcomes; limited colonization	Moderate
Prebiotics	Inulin, resistant starch, galacto-oligosaccharides	Non-living intervention; promotes endogenous beneficial microbes; favorable safety profile	Variable response depending on baseline microbiota composition	Moderate
Synbiotics	Combined probiotics and prebiotics	Synergistic microbiota modulation; enhanced SCFA production	Formulation heterogeneity; lack of standardized protocols	Moderate
Next-generation probiotics	Faecalibacterium prausnitzii, Akkermansia muciniphila	Targeted immunometabolic effects; strong mechanistic rationale	Manufacturing complexity; stability and regulatory challenges	Low–moderate
Fecal microbiota transplantation (FMT)	Healthy donor stool transplantation	Broad restoration of microbial diversity and function	Donor variability; pathogen transmission; regulatory concerns	Low
Defined microbial consortia	SER-109 and related live biotherapeutic products	Standardized composition; improved reproducibility and safety	Limited clinical experience in sepsis; manufacturing costs	Moderate
SCFA supplementation	Butyrate, acetate, propionate	Direct replacement of beneficial metabolites; well-defined mechanisms	Poor bioavailability; short half-life; delivery challenges	Moderate
Bile acid-based therapies	Ursodeoxycholic acid; FXR/TGR5 modulators	Targeted host signaling pathways; potential organ-protective effects	Metabolic side effects; limited clinical validation	Low–moderate
AhR ligand supplementation	Indole derivatives; tryptophan metabolites	Barrier protection; immune regulation	Pharmacokinetic uncertainty; optimal compounds not established	Low
Postbiotics	Purified microbial metabolites and bioactive products	Improved standardization; no risk of live microbial transmission	Limited clinical data; optimal dosing unknown	Moderate
Precision microbiome medicine	Microbiota-guided patient stratification and personalized interventions	Potential for individualized therapy and improved efficacy	Requires validated biomarkers, multi-omics integration, and prospective trials	Emerging

*Translational readiness reflects the current maturity of evidence supporting clinical development and implementation in sepsis, based on available preclinical studies, clinical trials, safety data, manufacturing feasibility, and regulatory considerations. AhR, aryl hydrocarbon receptor; FMT, fecal microbiota transplantation; FXR, farnesoid X receptor; SCFA, short-chain fatty acid; TGR5, Takeda G protein-coupled receptor 5.

## 5 The specific role of the gut-organ axis in sepsis-related multi-organ dysfunction

### 5.1 Gut-lung axis and acute respiratory distress syndrome (ARDS)

The gut–lung axis has emerged as an important mechanism linking intestinal dysbiosis to pulmonary inflammation and injury during sepsis and acute respiratory distress syndrome (ARDS). One proposed pathway involves the translocation of gut-derived bacteria, pathogen-associated molecular patterns (PAMPs), and lipopolysaccharides (LPS) across a compromised intestinal barrier into the systemic circulation, where they can activate pulmonary immune and endothelial cells, thereby amplifying lung inflammation and tissue injury (Dickson, 2016; Wang et al., 2025; Ye et al., 2025). In addition, disruption of intestinal barrier integrity may facilitate dissemination of microbial products through both portal and lymphatic pathways, contributing to systemic inflammatory responses that affect distant organs, including the lungs (Budden et al., 2017; Dickson, 2016).

Microbiota-derived metabolites also play an important role in gut–lung communication. Short-chain fatty acids (SCFAs), particularly butyrate and propionate, can modulate macrophage polarization, regulate neutrophil recruitment, and influence cytokine production, thereby affecting pulmonary immune homeostasis and susceptibility to lung injury (Dang and Marsland, 2019; Wypych et al., 2019). Experimental studies have suggested that depletion of SCFA-producing bacteria may exacerbate inflammatory responses in the lung, whereas restoration of microbial metabolic function may attenuate pulmonary inflammation (Dang and Marsland, 2019).

Furthermore, gut dysbiosis may contribute to ARDS through broader immunological mechanisms, including alterations in systemic immune responses, disruption of mucosal immunity, and dysregulated host–microbiome interactions (Dickson, 2016; Ye et al., 2025). Emerging evidence indicates that microbial signals originating from the intestine may influence pulmonary immune cell function and susceptibility to secondary respiratory infections, although direct clinical evidence in adult sepsis-associated ARDS remains limited (Enaud et al., 2020; Ye et al., 2025).

**FIGURE 5:**
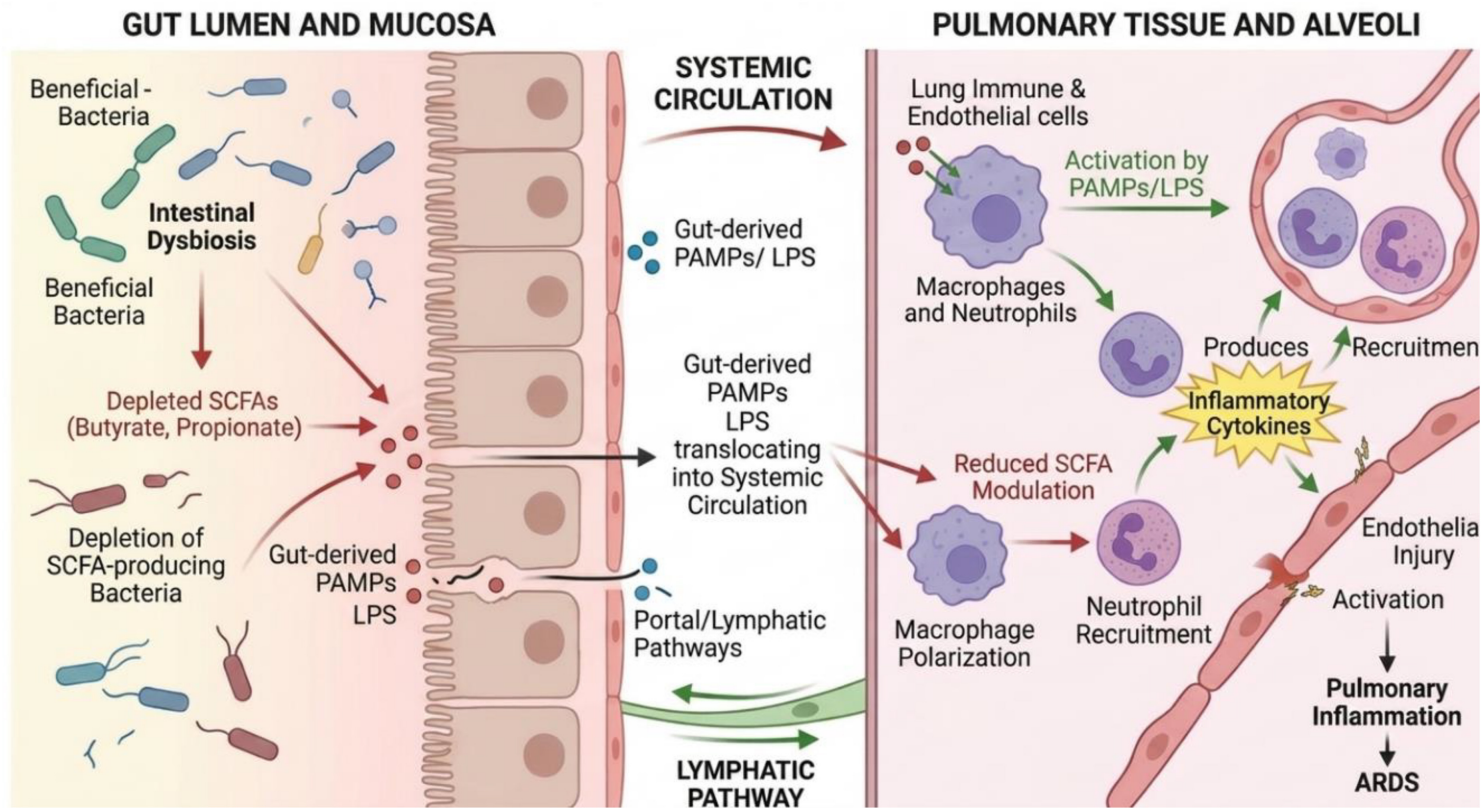
The gut–lung axis and its role in acute respiratory distress syndrome (ARDS) during sepsis. This figure illustrates the interactions between the gut microbiota and the lungs in the context of sepsis. Intestinal dysbiosis and barrier dysfunction lead to the translocation of microbial products and inflammatory mediators into the systemic circulation. These circulating factors can reach the lungs and contribute to pulmonary inflammation, immune dysregulation, and increased vascular permeability. In turn, lung injury and systemic inflammation may further alter gut microbiota composition and impair intestinal barrier function, establishing a bidirectional gut–lung axis. This interaction may play a role in the development and progression of sepsis-associated acute respiratory distress syndrome (ARDS). Overall, this figure highlights the systemic impact of gut microbiota dysbiosis and underscores the importance of gut–lung crosstalk in sepsis pathophysiology. ARDS, acute respiratory distress syndrome.

Collectively, these findings support the concept that intestinal dysbiosis is not merely a consequence of critical illness but may actively contribute to pulmonary injury through the gut–lung axis. Future studies should further clarify the mechanistic pathways involved and determine whether microbiota-targeted interventions can improve respiratory outcomes in patients with sepsis-associated ARDS (Figure 5).

### 5.2 Gut-brain axis and sepsis-associated encephalopathy (SAE)

Sepsis-associated encephalopathy (SAE) is one of the most common neurological complications of sepsis and is characterized by acute cognitive dysfunction, delirium, and long-term neurocognitive impairment. Increasing evidence suggests that the gut–brain axis plays a critical role in the pathogenesis of SAE by linking intestinal dysbiosis, systemic inflammation, and neuroinflammatory responses (Gofton and Young, 2012; He et al., 2025; Luo et al., 2024).

Disruption of intestinal barrier integrity during sepsis facilitates the translocation of microbial products, endotoxins, and inflammatory mediators into the systemic circulation. These factors can subsequently affect the central nervous system by activating microglia, promoting neuroinflammation, and impairing blood–brain barrier (BBB) integrity (Gofton and Young, 2012; Luo et al., 2024; Morais et al., 2021). Experimental studies have demonstrated that gut dysbiosis is associated with increased production of pro-inflammatory cytokines, altered neurotransmitter metabolism, and enhanced microglial activation, all of which contribute to neuronal injury and cognitive dysfunction (Luo et al., 2024; Sharon et al., 2016).

Microbiota-derived metabolites also play a central role in gut–brain communication. Short-chain fatty acids (SCFAs), tryptophan-derived metabolites, and other microbial products participate in the regulation of BBB function, neuroimmune signaling, and neuronal homeostasis (Dalile et al., 2019; Morais et al., 2021). Reduced production of these protective metabolites during sepsis may contribute to BBB disruption, neuroinflammation, and the development of delirium and cognitive impairment (Dalile et al., 2019).

Furthermore, emerging studies suggest that modulation of gut microbiota through dietary interventions, probiotics, prebiotics, and microbiota-targeted therapies may attenuate neuroinflammation and improve neurological outcomes in experimental models of SAE (Cryan et al., 2019; He et al., 2025). Although clinical evidence remains limited, these findings support the concept that restoration of microbiota homeostasis may represent a novel therapeutic strategy for preventing or mitigating SAE.

**FIGURE 6:**
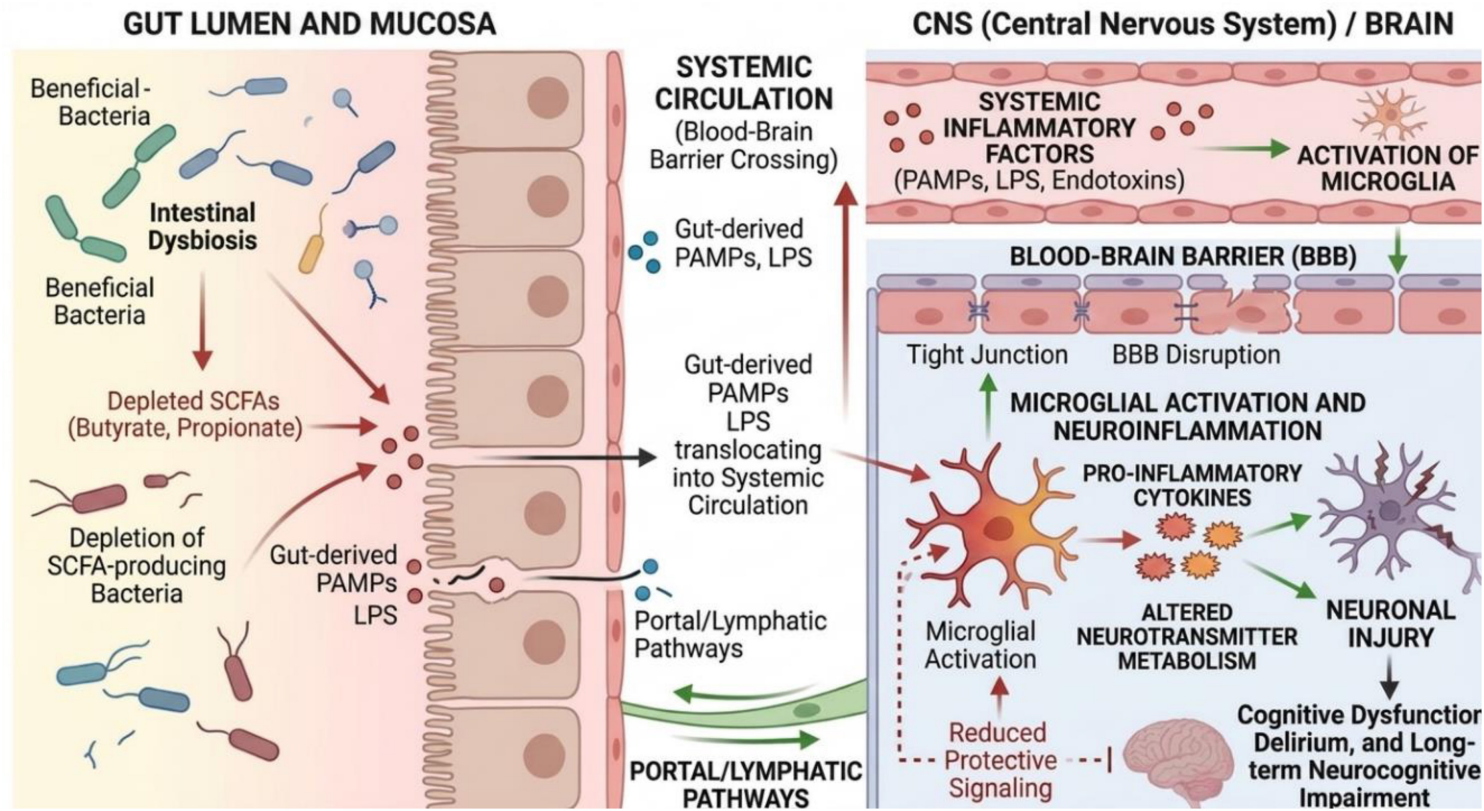
The gut–organ axis in sepsis: interactions with the lung and brain. This figure depicts the bidirectional interactions between the gut microbiota and distant organs, particularly the lungs and brain. Gut microbiota dysbiosis and barrier dysfunction lead to the release of microbial products and inflammatory mediators into the circulation, which can contribute to lung inflammation (gut–lung axis) and neuroinflammation (gut–brain axis). These pathways may increase susceptibility to sepsis-associated acute respiratory distress syndrome (ARDS) and sepsis-associated encephalopathy (SAE), highlighting the systemic impact of gut microbiota alterations. ARDS, acute respiratory distress syndrome; SAE, sepsis-associated encephalopathy.

Collectively, the gut–brain axis provides an important mechanistic framework linking intestinal dysbiosis to neurological dysfunction during sepsis. Future studies integrating microbiome profiling, metabolomics, and neuroimaging may help identify novel biomarkers and therapeutic targets for SAE (Figure 6; Sharon et al., 2016).

## 6 Current challenges in clinical translation

### 6.1 Individualization and timing of microbiota interventions

The implementation of microbiota-targeted therapies in sepsis faces substantial challenges owing to the marked interindividual variability of the gut microbiome. Factors including age, comorbidities, nutritional status, prior antibiotic exposure, and disease severity contribute to considerable differences in baseline microbial composition and function among septic patients (Krautkramer et al., 2021; Zmora et al., 2018). Consequently, a universal therapeutic approach may not be appropriate, as responses to probiotics, synbiotics, fecal microbiota transplantation (FMT), or metabolite-based therapies are likely to depend on the pre-existing microbiome configuration and host immune status.

Recent advances in metagenomics, metabolomics, and multi-omics integration have created opportunities for precision microbiome medicine. Future therapeutic strategies may incorporate microbiome profiling to identify patient-specific dysbiosis patterns and guide individualized interventions based on microbial composition, functional capacity, and metabolite production (Krautkramer et al., 2021; Lloyd-Price et al., 2016; Sharon et al., 2016). Such approaches could improve therapeutic efficacy while minimizing unnecessary or ineffective treatments.

In addition to patient stratification, the timing of intervention is likely to be a critical determinant of clinical success. The biological effects of microbiota modulation may vary across different stages of sepsis. During the early hyper-inflammatory phase, therapeutic strategies may focus on preserving intestinal barrier integrity, reducing microbial translocation, and limiting excessive inflammatory responses. In contrast, interventions administered during later immunosuppressive phases may aim to restore microbial diversity, improve immune competence, and reduce susceptibility to secondary infections (Haak et al., 2023; Shimizu et al., 2025; Wischmeyer et al., 2023).

Despite increasing recognition of these concepts, robust evidence defining optimal treatment windows remains lacking. Most clinical studies have been limited by heterogeneous patient populations, variable intervention protocols, and insufficient longitudinal microbiome monitoring (Lou et al., 2024; Ojima et al., 2016). Furthermore, several ongoing clinical trials are currently evaluating microbiota-targeted therapies in critically ill patients, highlighting growing interest in translating microbiome-based interventions into clinical practice (Chamani et al., 2024).

Future large-scale randomized controlled trials incorporating serial microbiome analyses, host immune profiling, and clinical phenotyping will be required to establish optimal timing strategies and validate personalized microbiota interventions for sepsis management (Durack and Lynch, 2019; Krautkramer et al., 2021; Zmora et al., 2018).

### 6.2 Standardization of research methodology and outcome measures

The advancement of microbiota research in sepsis is currently limited by substantial methodological heterogeneity across studies. Variations in sample collection procedures, storage conditions, DNA extraction methods, sequencing platforms, bioinformatic pipelines, and statistical analyses can significantly influence microbiome profiles and contribute to inconsistent findings (Knight et al., 2018; Pollock et al., 2018; Sinha et al., 2017). These methodological differences reduce reproducibility and hinder meaningful comparisons across studies, thereby limiting the translation of microbiome research into clinical practice. Consequently, the establishment of standardized operating procedures and reporting frameworks for microbiota studies in sepsis should be considered a research priority.

In addition to technical standardization, greater consistency in study design and outcome assessment is required. Current investigations frequently employ heterogeneous clinical endpoints, making it difficult to compare intervention efficacy across studies. While mortality remains an important endpoint, it may not adequately capture the biological effects of microbiota-targeted therapies (Haak and Wiersinga, 2017; Sinha et al., 2017). Future studies should therefore incorporate more comprehensive outcome frameworks that integrate both clinical and microbiological measures.

Potential microbiota-related endpoints include restoration of microbial diversity, recovery of beneficial taxa, normalization of microbial metabolite profiles, improvement of intestinal barrier function, reduction in secondary infections, and duration of organ support (Haak and Wiersinga, 2017; Haak et al., 2023; Integrative HMP (iHMP) Research Network Consortium, 2019). Incorporating these intermediate biological outcomes may provide a more mechanistic assessment of therapeutic efficacy and facilitate interpretation of clinical trial results.

Furthermore, integration of metagenomics, metabolomics, transcriptomics, and host immune profiling may help establish standardized multi-omics biomarkers and improve comparability among studies (Integrative HMP (iHMP) Research Network Consortium, 2019; Sharon et al., 2016). The development of consensus outcome sets, standardized analytical pipelines, and internationally harmonized reporting guidelines will be essential for advancing microbiota-based precision medicine in sepsis (Knight et al., 2018; Zmora et al., 2018).

## 7 Future research directions and outlook

### 7.1 Development of AI-based precision diagnosis and treatment platforms

Artificial intelligence (AI) and machine learning technologies are increasingly recognized as powerful tools for analyzing the complex and multidimensional datasets generated by microbiome research. In sepsis, AI-based approaches may facilitate the integration of microbial taxonomic profiles, functional metagenomic data, metabolomic signatures, host immune parameters, and clinical variables to improve disease characterization and therapeutic decision-making (Franzosa et al., 2019; Knights et al., 2011; Topçuoğlu et al., 2020).

Recent studies have demonstrated the potential of machine learning algorithms to identify microbiota-based biomarkers, classify disease phenotypes, predict clinical outcomes, and support risk stratification in critically ill patients (Franzosa et al., 2019; Li et al., 2023; Yagin et al., 2024). In particular, multi-omics integration platforms combining microbiome sequencing, metabolomics, transcriptomics, and clinical data may enable more comprehensive assessment of host–microbiome interactions and provide a foundation for precision medicine approaches in sepsis (Integrative HMP (iHMP) Research Network Consortium, 2019; Sharon et al., 2016; Topçuoğlu et al., 2020).

Beyond diagnostic applications, AI-driven analytical frameworks may also facilitate personalized microbiota-targeted interventions. Predictive models could potentially identify patients most likely to benefit from probiotics, synbiotics, fecal microbiota transplantation (FMT), metabolite-based therapies, or other microbiome-directed treatments based on individual microbiome characteristics and host immune profiles (Knights et al., 2011; Krautkramer et al., 2021; Zmora et al., 2018). Such approaches may help optimize patient selection and improve therapeutic efficacy while reducing unnecessary interventions.

Despite these promising developments, significant challenges remain. Current machine learning models are frequently limited by small sample sizes, cohort heterogeneity, lack of external validation, and inconsistencies in microbiome data generation and analysis (Knight et al., 2018; Wirbel et al., 2021). Furthermore, issues related to model interpretability, data standardization, regulatory oversight, and clinical implementation must be addressed before AI-assisted microbiome medicine can be routinely integrated into sepsis management (Beam and Kohane, 2018).

Future research should focus on establishing large multicenter datasets, standardized analytical pipelines, and externally validated predictive models to facilitate the development of AI-based precision diagnosis and treatment platforms for sepsis.

### 7.2 Exploration of next-generation live biotherapeutic products (lBPs)

The development of live biotherapeutic products (LBPs), defined as biologically active microorganisms intended for therapeutic use, represents an emerging frontier in microbiome-based medicine. Unlike conventional probiotics, LBPs are typically composed of highly characterized microbial strains or defined microbial consortia specifically selected for their therapeutic functions (Charbonneau et al., 2020; O’Toole et al., 2017). These next-generation microbiome therapies are designed to exert targeted biological effects, including restoration of microbial ecosystem balance, enhancement of intestinal barrier integrity, modulation of host immune responses, and sustained production of beneficial microbial metabolites.

Recent advances in microbial engineering and synthetic biology have further expanded the therapeutic potential of LBPs. Genetically engineered microorganisms may be designed to produce anti-inflammatory metabolites, deliver therapeutic molecules, modulate immune signaling pathways, or selectively suppress pathogenic microorganisms (Riglar and Silver, 2018; Rouanet et al., 2020). Such approaches offer opportunities to develop more precise and reproducible microbiome-based interventions compared with traditional probiotic formulations.

Despite their considerable promise, the development of LBPs for sepsis remains at an early stage. Several major challenges must be addressed before clinical implementation can be achieved. These include ensuring long-term colonization stability, maintaining manufacturing consistency, establishing scalable production processes, optimizing delivery systems, and evaluating potential safety concerns related to microbial persistence, horizontal gene transfer, and unintended host–microbiome interactions (Cordaillat-Simmons et al., 2020; O’Toole et al., 2017; Riglar and Silver, 2018). Furthermore, regulatory frameworks for LBPs continue to evolve, creating additional challenges for clinical translation and commercialization.

**FIGURE 7:**
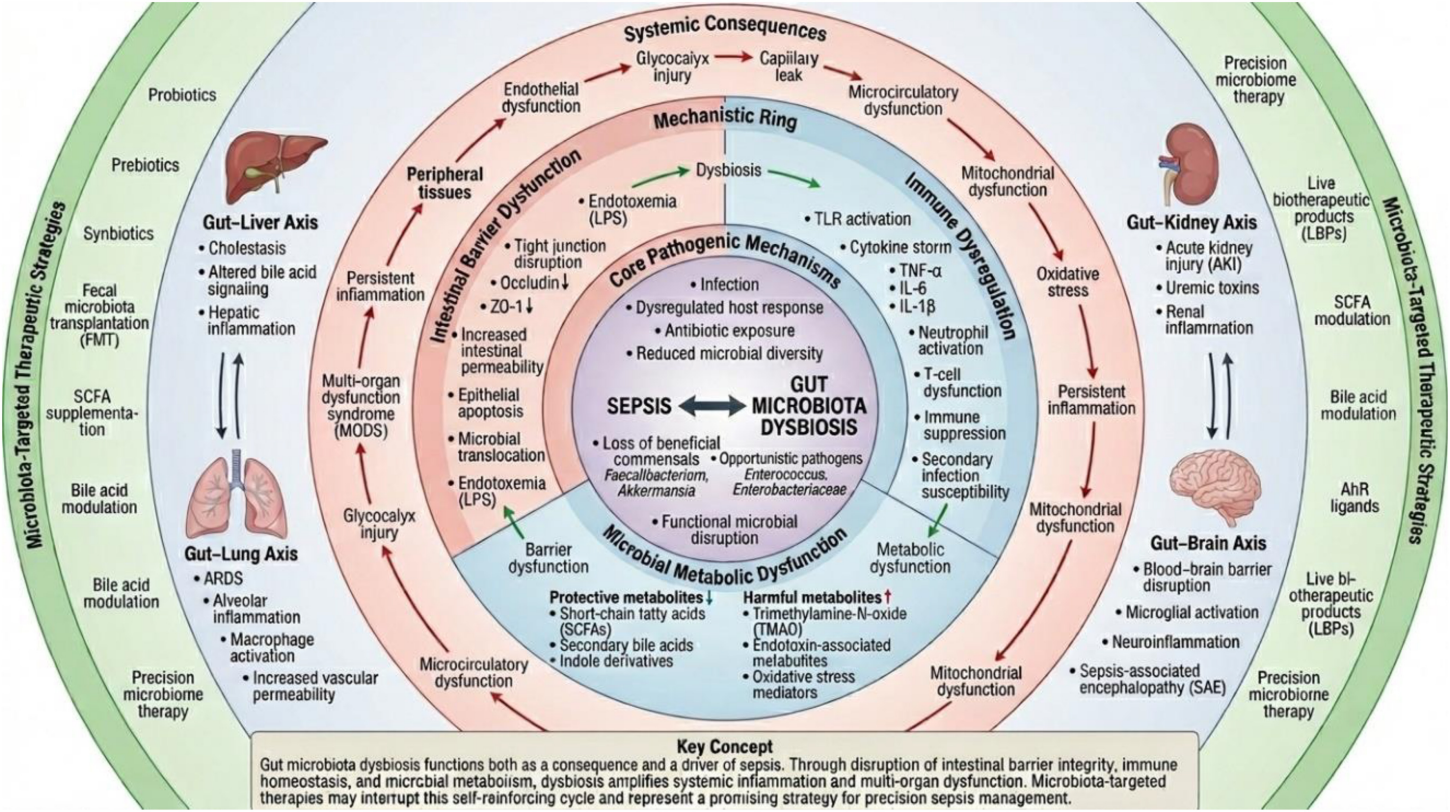
Bidirectional interactions between sepsis and gut microbiota dysbiosis: pathogenic mechanisms, organ crosstalk, and therapeutic opportunities. Sepsis and gut microbiota dysbiosis form a self-amplifying cycle driven by intestinal barrier dysfunction, immune dysregulation, and microbial metabolic dysfunction. These interconnected mechanisms contribute to systemic inflammation, endothelial injury, microcirculatory dysfunction, oxidative stress, and multi-organ dysfunction. Gut microbiota alterations further influence distant organs through the gut–lung, gut–brain, gut–liver, and gut–kidney axes. Emerging microbiota-targeted therapeutic strategies, including probiotics, prebiotics, synbiotics, fecal microbiota transplantation, microbial metabolite modulation, live biotherapeutic products, and precision microbiome-based approaches, may help restore microbial homeostasis and interrupt this pathogenic cycle.

At present, LBPs should be regarded as promising but largely investigational therapeutic candidates in the context of sepsis. Future studies should focus on validating these approaches in sepsis-specific experimental models and clinical cohorts, identifying optimal patient populations, defining treatment timing, and integrating microbiome profiling with precision medicine strategies (Charbonneau et al., 2020; Hwang et al., 2017; Rouanet et al., 2020). The major mechanisms, systemic consequences, gut–organ interactions, and microbiota-targeted therapeutic opportunities discussed throughout this review are summarized in Figure 7.

### 7.3 In-depth analysis of microbiome-host interactions at the molecular level

A deeper understanding of the molecular mechanisms governing host–microbiome interactions will be essential for advancing microbiota-based therapies in sepsis. Although substantial progress has been made in characterizing alterations in microbial composition and metabolite profiles, the precise pathways through which gut microorganisms influence host immunity, barrier function, and systemic organ responses remain incompletely understood (Lynch and Pedersen, 2016; Walter et al., 2020).

Future investigations should increasingly utilize germ-free and gnotobiotic animal models to dissect causal relationships between specific microbial taxa, microbial metabolites, and host physiological responses during sepsis (Faith et al., 2010; Walter et al., 2020). These experimental systems provide unique opportunities to identify microbial species and metabolic pathways that regulate immune homeostasis, intestinal barrier integrity, and susceptibility to organ dysfunction.

Emerging technologies, including organ-on-chip platforms and advanced *in vitro* co-culture systems, may further facilitate mechanistic studies of host–microbiome interactions under controlled conditions (Bein et al., 2018). Such approaches may help clarify how microbial signals influence intestinal epithelial cells, intestinal stem cells, Paneth cells, goblet cells, and other specialized cell populations involved in mucosal homeostasis and host defense (Allaire et al., 2018; Kayama et al., 2020). In addition, increasing evidence suggests that gut microbiota may influence hematopoiesis, innate immune memory (trained immunity), and peripheral immune cell programming, thereby affecting systemic responses to infection and critical illness (Netea et al., 2020).

The integration of multi-omics technologies, including metagenomics, transcriptomics, proteomics, metabolomics, and single-cell sequencing, is expected to provide a more comprehensive understanding of host–microbiome dynamics during sepsis (Hasin et al., 2017; Integrative HMP (iHMP) Research Network Consortium, 2019; Sharon et al., 2016). Such systems-level approaches may facilitate identification of novel signaling pathways, therapeutic targets, and predictive biomarkers while enabling construction of increasingly sophisticated models of microbiome–host interactions.

Ultimately, combining mechanistic experimental studies with multi-omics integration and computational modeling may accelerate the development of precision microbiome therapeutics and improve our understanding of how microbial ecosystems influence susceptibility, progression, and recovery in sepsis.

## 8 Conclusion

Accumulating evidence indicates that gut microbiota dysbiosis is not merely a consequence of sepsis but an active participant in its pathogenesis. Through disruption of intestinal barrier integrity, immune dysregulation, and alterations in microbiota-derived metabolites, gut microbiota contribute to systemic inflammation, organ dysfunction, and adverse clinical outcomes. These interconnected mechanisms support the concept that sepsis should be viewed not only as a dysregulated host response to infection but also as a disorder of host–microbiome interactions.

Recent advances in metagenomics, metabolomics, and microbiome research have identified gut microbial signatures and microbiota-derived metabolites as promising candidates for diagnostic and prognostic biomarkers. In parallel, microbiota-targeted interventions—including probiotics, prebiotics, fecal microbiota transplantation, microbial metabolite supplementation, and emerging live biotherapeutic products—have demonstrated encouraging therapeutic potential in preclinical studies and early clinical investigations. Collectively, these findings suggest that modulation of the gut microbiota may represent a novel adjunctive strategy for improving outcomes in sepsis.

Nevertheless, substantial challenges remain before microbiome-based approaches can be incorporated into routine clinical practice. Inter-individual variability in microbiota composition, differences in sample collection and analytical methodologies, heterogeneous clinical trial designs, and limited reproducibility across populations continue to hinder clinical translation. Furthermore, the optimal timing, patient selection criteria, and long-term safety of microbiota-targeted interventions remain incompletely defined.

Future progress in this field will require large multicenter longitudinal studies, standardized microbiome profiling protocols, and integration of multi-omics technologies, including metagenomics, transcriptomics, metabolomics, and proteomics. Advances in artificial intelligence and machine learning may facilitate the identification of clinically relevant microbiome signatures and support precision risk stratification. Ultimately, the development of personalized microbiota-targeted therapies based on individual microbial and metabolic profiles may provide new opportunities for precision medicine in sepsis.

In summary, growing recognition of the gut microbiota as a key regulator of sepsis pathophysiology has transformed our understanding of this complex syndrome. Continued efforts to bridge mechanistic discoveries with clinical translation are expected to accelerate the development of microbiome-informed diagnostic and therapeutic strategies, with the potential to improve survival and long-term outcomes for patients with sepsis.
